# A novel long non-coding natural antisense RNA is a negative regulator of *Nos1* gene expression

**DOI:** 10.1038/srep11815

**Published:** 2015-07-08

**Authors:** Sergei A. Korneev, Mark Maconochie, Souvik Naskar, Elena I. Korneeva, Guy P. Richardson, Michael O’Shea

**Affiliations:** 1 Sussex Neuroscience, School of Life Science, University of Sussex, Brighton BN1 9QG, UK; 2 School of Biological and Chemical Sciences, Queen Mary University of London, London E1 4NS, UK

## Abstract

Long non-coding natural antisense transcripts (NATs) are widespread in eukaryotic species. Although recent studies indicate that long NATs are engaged in the regulation of gene expression, the precise functional roles of the vast majority of them are unknown. Here we report that a long NAT (*Mm-antiNos1* RNA) complementary to mRNA encoding the neuronal isoform of nitric oxide synthase (Nos1) is expressed in the mouse brain and is transcribed from the non-template strand of the *Nos1* locus. Nos1 produces nitric oxide (NO), a major signaling molecule in the CNS implicated in many important functions including neuronal differentiation and memory formation. We show that the newly discovered NAT negatively regulates *Nos1* gene expression. Moreover, our quantitative studies of the temporal expression profiles of *Mm-antiNos1* RNA in the mouse brain during embryonic development and postnatal life indicate that it may be involved in the regulation of NO-dependent neurogenesis.

Natural antisense transcripts (NATs) are endogenous RNA molecules that are complementary to RNA transcripts of already established function. The vast majority of NATs are not translated into proteins and therefore belong to the category of non-coding RNAs. Depending on their origin, all NATs belong to one of two main types: *cis*-encoded and *trans*-encoded. *Cis*-encoded NATs (*cis*NATs) are produced from the same loci as their sense counterparts whereas *trans*-encoded NATs (*trans*NATs) are transcribed from different loci. Until recently, NATs in eukaryotic systems were thought to be a minor group of unimportant transcripts of no known function. But it is now quite clear that NATs are far more abundant in eukaryotic systems than previously thought^1^,^2^,^3^,^4^,^5^. Indeed they appear to be especially prevalent in the nervous system, suggesting they may have important functions in the brain.

This view is supported by our experiments conducted on the molluscan model system *Lymnaea stagnalis*, in which we identified neuronal NATs complementary to mRNA encoding the nitric oxide synthase (NOS), the enzyme responsible for production of the endogenous gaseous neurotransmitter nitric oxide (NO)^6^. We have demonstrated that these NATs play an important role in the regulation of the NO signalling – a pathway required for the formation of long-term memory^7^,^8^,^9^. Recent complementary studies in mammals indicate the existence of similar NAT-mediated mechanisms regulating NO production. In mammals, NO is implicated in many different biological functions^10^,^11^,^12^,^13^,^14^ and is produced by three major NOS isoforms: neuronal (NOS1), inducible (NOS2) and endothelial (NOS3). Notably, NATs complementary to NOS2- and NOS3-encoding mRNAs nave been recently identified and characterised^15^,^16^,^17^. For the *NOS1* gene, which encodes the key enzyme producing NO in the brain (for a review, see 18), there is as yet no evidence concerning NAT-mediated regulation of its expression.

Here we report on the discovery of a NAT, which is expressed in the mouse brain and is complementary to the *Nos1* mRNA. We named this transcript *Mm-antiNOS1* RNA and show that it belongs to a class of *cis*NATs. We demonstrate that the *Mm-antiNOS1* RNA acts as a negative regulator of *Nos1* gene expression. Our data also suggest that *Mm-antiNOS1* RNA is involved in the regulation of neurogenesis.

## Results

### *A cis*-encoded NAT complementary to *Nos1* mRNA is expressed in the mouse brain

**Figure 1:**
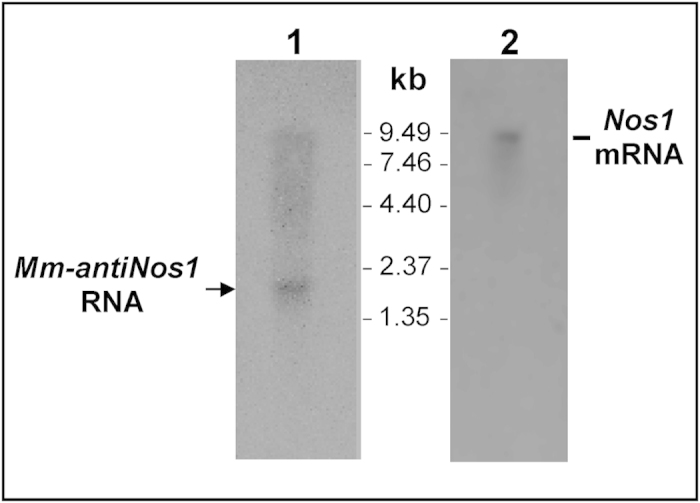
*Nos1*-related natural antisense RNA (*Mm-antiNos1* RNA) is expressed in the mouse brain. In 1, Northern blot analysis of poly(A)^+^ RNA extracted from the mouse brain using the ‘sense’ probe. The ‘sense’ probe has the same sequence as *Nos1* mRNA. A prominent band (shown by arrow) indicates the presence of endogenous antisense transcript of approximately 1500 nt. In 2, The result of Northern blot hybridisation with the ‘antisense’ probe that is complementary to *Nos1* mRNA. As expected, a band of about 9 kb is revealed.

To establish whether a Nos1-related NAT is expressed in mice we first employed Northern blot hybridization. Approximately 3 μg of poly(A)^+^ RNA isolated from the mouse brain were used in this analysis. Two radiolabeled hybridization probes designated as ‘sense’ and ‘antisense’ were produced by asymmetrical PCR. The ‘sense’ probe had the same sequence as *Nos1* mRNA whereas the ‘antisense’ probe was complementary to *Nos1* mRNA. Notably, hybridization with the sense probe revealed a prominent band corresponding to a transcript of about 1500 nt in length indicative of the presence of a *Nos1*-related antisense RNA in the mouse brain (Fig. 1a). The control antisense probe detected a transcript of about 9000 nt (Fig. 1b) that was exactly the expected size for *Nos1* mRNA confirming the integrity of RNA used for the analysis.

**Figure 2:**
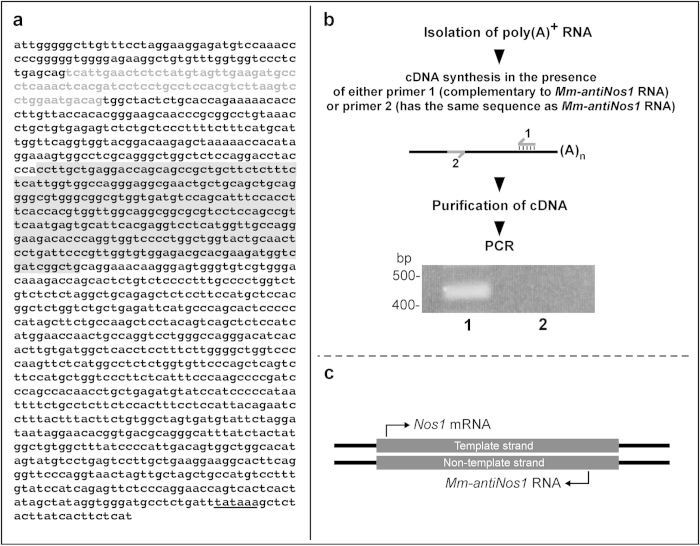
*Mm-antiNos1* RNA is a polyadenylated *cis*-encoded natural antisense transcript. (**a**) Nucleotide sequence of *Mm-antiNos1* RNA. The antisense region is shaded. A putative polyadenylation signal is underlined. The alternatively spliced exon is shown in grey letters. (**b**) The results of RT-PCR experiment performed on poly(A)^+^ RNA extracted from the mouse brain. A product of the expected size (about 0.45 kb) is produced when primer 1 was used in the reverse transcription reaction (lane 1). Note that there is no product in the experiment in which primer 2 was used (lane 2). (**c**) A schematic diagram showing that *Mm-antiNos1* RNA is transcribed from the non-template strand of the *Nos1* locus.

To determine the primary structure of the antisense RNA detected by the Northern blot hybridization we conducted a BLAST search of the NCBI nucleotide database. This has revealed a non-coding sequence (AK146140) containing a region complementary to the *Nos1* mRNA (Fig. 2a). The putative antisense region of the AK146140 is about 300 nt and exhibits 100% complementarity to a region located within the open reading frame of the *Nos1* mRNA. The remaining part of the AK146140 is identical to intron sequences of the *Nos1* gene, indicating that AK146140 is a spliced transcript. Taken together these results suggest that AK146140 represents a *Nos1*-related NAT produced from the non-template strand of the *Nos1* locus.

Although the AK146140 contains a putative polyadenylation signal at an appropriate position from the 3’ end, it does not contain a poly(A) tail. Therefore it was important to verify the true orientation of the sequence deposited in the database. This was achieved by RT-PCR using poly(A)^+^ RNA as a template. Two reverse transcription reactions were set up. In the first reaction a primer complementary to AK146140 (primer 1) was used. In the second reaction a primer that had the same sequence as AK146140 was used (primer 2). The products of the reactions were then purified to remove cDNA primers and the purified cDNAs were subjected to PCR in the presence of primers internal to the position of primers used in the reverse transcription reaction. The results of the analysis shown in Fig. 2b demonstrate that the PCR product of exactly the expected size is detected only when primer 1 was used. This shows that the primary structure of AK146140 corresponds to a real polyadenylated RNA and justifies the conclusion that a non-coding *Nos1*-related *cis*NAT is expressed in the mouse brain (Fig. 2c). Hereafter we will refer to this transcript as *Mm-antiNos1* RNA.

### *Mm-antiNos1* RNA down-regulates Nos1 protein synthesis in Neuro2a cells

Our discovery that a *Nos1*-related *cis*NAT is expressed in the mouse brain raised the intriguing possibility that this non-coding RNA is involved in the regulation of *Nos1* gene expression. To verify this suggestion we used Neuro2a neuroblastoma cells, a mouse neural crest-derived cell line. Importantly, we found that Neuro2a cells normally express both *Mm-antiNos1* RNA and *Nos1* mRNA and the levels of their expression in Neuro2a are comparable to those observed in the mouse brain (data not shown).

**Figure 3:**
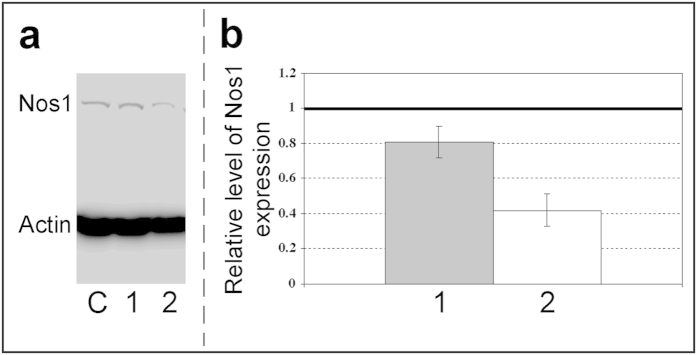
*Mm-antiNos1* RNA down-regulates Nos1 protein synthesis. (**a**) Representative Western blot analysis of cultured Neuro2a cells incubated with transfection mixtures containing either 0.2 μg (lane 1) or 1 μg (lane 2) of pcDNA 3.1/Mm-antiNos1. Non-transfected control cells are shown in lane C. (**b**) Quantitative densitometric analysis of the Western blot shown in a. Values for the Nos1 expression were quantified relative to the expression of β-actin. Finally, the values for Nos1 expression in transfected Neuro2a cells were normalised to Nos1 protein levels in non-transfected cells.

To examine the impact of *Mm-antiNos1* RNA on *Nos1* gene expression we overexpressed this NAT in Neuro2a cells. First we engineered an expression construct containing the full length *Mm-antiNos1* cDNA. We then transiently transfected Neuro2a cells with this *Mm-antiNos1* expression construct using Lipofectamine 2000. Success of the transfection experiments has been confirmed by the results of real-time RT-PCR showing significant up-regulation of *Mm-antiNos1* RNA in the transfected cells (data not shown). It is of importance that the quantitative analysis did not detect any changes in the expression level of *Nos1* mRNA in cells overexpressing *Mm-antiNos1* RNA (data not shown). Remarkably, however, our experiments showed that the overexpression of *Mm-antiNos1* RNA caused a substantial reduction of Nos1 protein produced (Fig. 3). Thus we can conclude that *Mm-antiNos1* RNA negatively regulates *Nos1* gene expression and does so post-transcriptionally.

### Expression profiles of *Mm-antiNos1* RNA and *Nos1* mRNA indicate a developmental role

**Figure 4:**
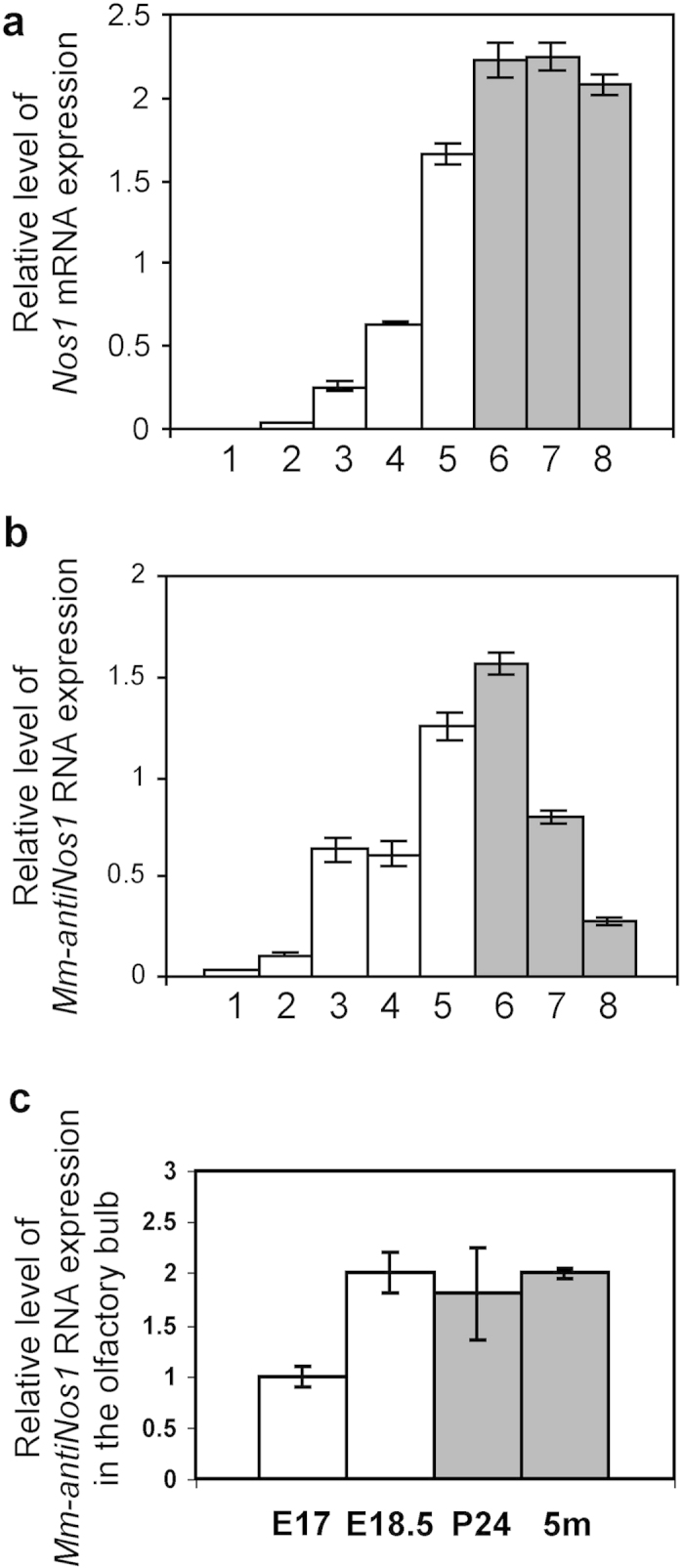
The expression profiles of *Nos1* mRNA and *Mm-antiNos1* RNA during embryonic development and postnatal life. (**a**) Results of real-time RT-PCR analysis of *Nos1* mRNA expression. (**b**) Results of real-time RT-PCR analysis of *Mm-antiNos1* RNA expression. Real-time RT-PCR experiments have been performed on individual brains dissected at different stages of embryonic development (white bars) and postnatal life (grey bars): 1 - E9.5, 2 - E11.5, 3 - E13.5, 4 - E15.5, 5 - E18.5, 6 - P1, 7 - P20, 8 - 4 months. (**c**) Results of real-time RT-PCR analysis of *Mm-antiNos1* RNA expression in the OBs dissected from embryonic (white bars) and adult (grey bars) brain. The values represent the average of three independent experiments.

Because NO signalling is known to be involved in the regulation of neurogenesis (for a review, see 19), we decided to determine whether there are any correlations between *Mm-antiNos1* RNA expression and embryonic and post-embryonic stages of development of the brain. To achieve this we extracted RNA from individual brains at 8 developmental time-points spanning embryonic (E9.5, E11.5, E13.5, E15.5, E18.5) and post-natal (P1, P20) phases and adulthood (4 months). The RNA extracts were then subjected to real-time RT-PCR. The results of the analysis show that the expression of both *Mm-antiNos1* RNA and *Nos1* mRNA is gradually increased during embryogenesis (Fig. 4). However, in the adult brain the expression level of *Nos1* mRNA remains quite stable until mice are at least 4 months old, whereas the expression level of *Mm-antiNos1* RNA is decreased quite rapidly (Fig. 4). These results are interesting because they demonstrate that the up-regulation of *Mm-antiNos1* RNA coincides with phases of extensive neurogenesis in the embryo. Furthermore, the age-related decline in neurogenesis in the adult mouse brain is associated with down-regulation of *Mm-antiNos1* RNA expression. There appears therefore to be a strong association between fluctuating rates of neurogenesis and the expression of *Mm-antiNos1* RNA.

While rapid and pervasive neurogenesis is a feature principally of the embryonic brain, significant neurogenesis does, however, occur in restricted regions of the adult brain. If the association between neurogenesis and *Mm-antiNos1* RNA expression is of functional significance one would predict that in spite of the overall decline in Mm-antiNos1 RNA expression in aged mice, high levels of expression would be retained in those regions of the brain in which neurogenesis persists into adulthood. To examine this prediction we quantified the profile of *Mm-antiNos1* RNA expression in the olfactory bulb (OB), a region of active neurogenesis in adulthood. We carried out real-time RT-PCR analysis on the OBs dissected from the embryonic (E17 and E18.5), post-natal (P24), and adult (5 months) brain. We found that the expression of *Mm-antiNos1* is increased during embryogenesis (Fig. 4c) and does not decline until mice are at least 5 months old. Thus these observations support our idea that active neurogenesis is associated with up-regulation of *Mm-antiNos1* RNA.

### *Mm-antiNos1* RNA is up-regulated during differentiation of Neuro2a cells into neurons

**Figure 5:**
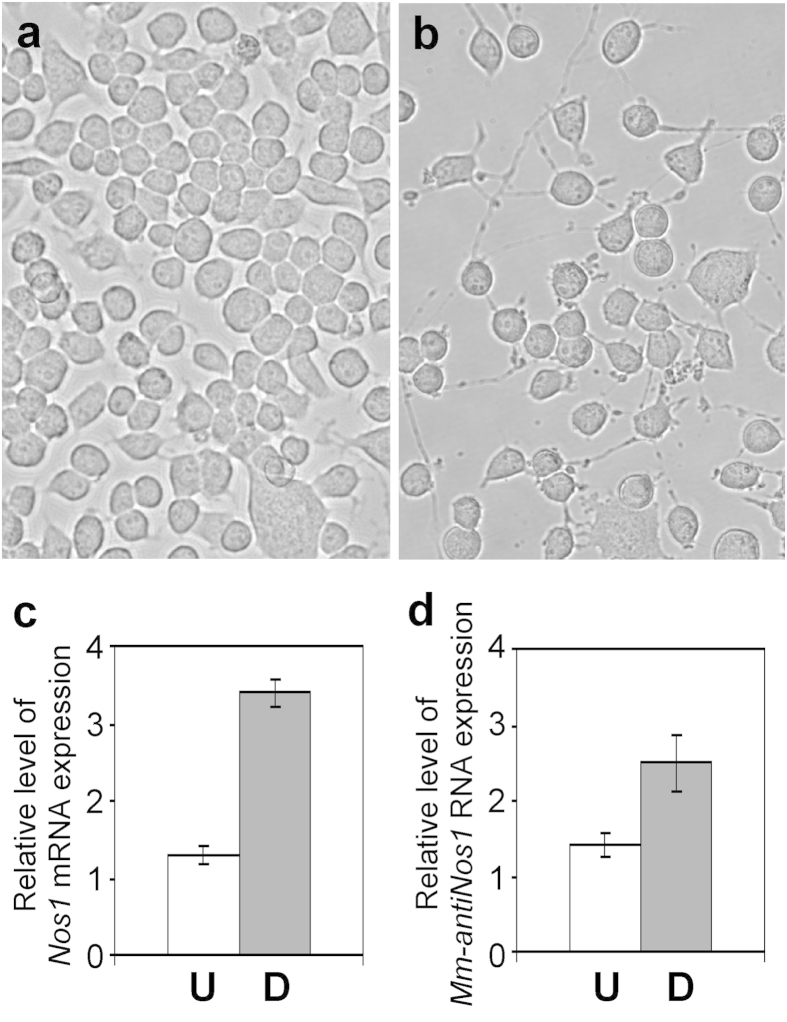
Quantitative analysis of *Nos1* mRNA and *Mm-antiNos1* RNA expression during neuronal differentiation of Neuro2a cells. (**a**) Undifferentiated Neuro2a cells. (**b**) Neuro2a cells differentiated for 4 days in low-serum media with retinoic acid. (**c**) Results of real-time RT-PCR analysis of *Nos1* mRNA expression: U – undifferentiated Neuro2a cells, D – differentiated Neuro2a cells. (**d**) Results of real-time RT-PCR analysis of *Mm-antiNos1* RNA expression: U – undifferentiated Neuro2a cells, D – differentiated Neuro2a cells. The values represent the average of three independent experiments.

Our experiments *ex vivo* (see Fig. 4) suggested that characteristic changes in the expression of *Mm-antiNos1* RNA and *Nos1* mRNA were associated with temporal dynamics of brain development and neurogenesis. As the Neuro2a cell line is a well-established model to study some aspects of neurogenesis, we employed this cell system to examine possible correlations between neuronal differentiation and changes in the expression level of *Mm-antiNos1* RNA. We used a combination of retinoic acid and reduced levels of serum in the culture medium to induce neuronal differentiation. Four days after induction the Neuro2a cells established a dense network of neurites (Fig. 5a,b). A comparison of undifferentiated and differentiated cells using real-time RT-PCR revealed that both *Nos1* mRNA and *Mm-antiNos1* RNA are up-regulated during neuronal differentiation (Fig. 5c,d). These data are reminiscent of our observations made *ex vivo* and add to the evidence that the *Nos1*-related NAT functions in the context of the role of NO signalling in the regulation of neurogenesis.

## Discussion

A number of studies have demonstrated that NO plays an important role in embryonic and adult neurogenesis by regulating neuronal differentiation, synapse formation and patterning^20^,^21^,^22^,^23^,^24^. For example NO produced by NOS1 inhibits proliferation of neural precursor cells and is involved in the regulation of a switch from cellular proliferation to neuronal differentiation^25^,^26^,^27^. In performing these functions, synthesis of NO must be tightly regulated because high concentrations of NO contribute to pathological conditions of the nervous system such as neuroinflammation and neurodegenerative disorders (for a review, see 28). Therefore NO production in the CNS must be tightly controlled. It appears that a variety of mechanisms, operating at different levels, exist to avoid the generation of damaging NO concentrations^29^,^30^. This paper presents evidence for a role of a *Nos1*-related NAT in the regulation of NO signaling.

Here we report that a long NAT complementary to the mRNA encoding Nos1 protein, the key enzyme producing NO in the CNS, is expressed in the mouse brain. This non-coding NAT, named *Mm-antiNos1* RNA, is transcribed from the non-template strand of the *Nos1* locus and therefore belongs to a class of *cis*NATs. These findings raised the intriguing possibility that *Mm-antiNos1* RNA could be involved in the regulation of neuronal NO signaling.

To address this important issue we employed an *in vitro* approach. By manipulating the expression of the *Mm-antiNos1* RNA in Neuro2a cells we examined the effect of this NAT on *Nos1* gene expression. A limited number of studies focusing on the functional roles of long NATs are available and they reveal both negative and positive effects on gene expression were reported^16^,^31^,^32^,^33^,^34^,^35^. Our experiments show that although over expression of the *Mm-antiNos1* RNA does not lead to a decrease in the amount of sense *Nos1* mRNA, it significantly down-regulates the amount of *Nos1* protein synthesised. Thus, we can conclude that *Mm-antiNos1* RNA is a post-transcriptional negative regulator of *Nos1* gene expression.

The temporal expression profile of *Mm-antiNos1* RNA in the mouse brain suggested a role for this NAT in the regulation of neurogenesis both in the developing embryo and during adulthood. *Mm-antiNos1* RNA expression, which is first observed at E9.5, increases gradually during embryogenesis and reaches its maximum level in newborn animals. In postnatal mice expression levels of the *Mm-antiNos1* RNA decline rapidly with age and by P20 there is a twofold drop in the NAT level compared to P1. As neurogenesis in mammals is restricted to embryonic and early postnatal stages and is very limited in adults, the up-regulation of *Mm-antiNos1* RNA coincides with high embryonic neurogenesis and down-regulation of this NAT is associated with the postnatal suppression of neurogenesis. An additional interesting issue emerges from our findings that the expression level of *Nos1* mRNA is also gradually increased in the brain during embryogenesis, but remains quite stable through postnatal stages and in adult mice until at least 4 months of age. Based on the findings described above, we can make the following inferences. Firstly, we suggest that *Mm-antiNos1* RNA works as a negative regulator of *Nos1* gene expression. Secondly, the gradually increased expression of *Mm-antiNos1* RNA observed during embryogenesis can compensate for the rapid up-regulation of *Nos1* gene allowing proliferation of neural stem cells in the embryonic brain. Thirdly, in adult mice the negative regulatory effect provided by *Mm-antiNos1* RNA diminishes and consequently the increased level of NO will inhibit neurogenesis.

Additional support for the proposed role of *Mm-antiNos1* RNA in the regulation of neurogenesis comes from our quantitative experiments in which we studied *Mm-antiNos1* RNA expression in the OBs isolated from the embryonic, post-natal, and adult brain. The rationale for this was that recent studies indicated that the OBs of adult mice are active sites of the late stages of neurogenesis^36^. Thus neurogenesis-associated changes in gene expression in the adult brain could be detected more easily in the OB than in the entire brain, in which the neurogenic regions constitute only a very small part. Also the OB can be unmistakably identified and dissected that minimises the risk of contamination by other brain structures. Notably, our experiments have shown that the expression level of *Mm-antiNos1* RNA in the OB remained at a relatively high level in both perinatal and adult mice. This is in direct contrast to what we observed at the whole-brain level where the *Mm-antiNos1* RNA declined rapidly in adults. Thus, we can see again that the up-regulation of *Mm-antiNos1* RNA is associated with active neurogenesis.

To further consolidate the functional role of *Mm-antiNos1* RNA, we also used an important property of Neuro2a cells to respond to serum deprivation and some specific stimuli (e.g., RA) by expressing signaling molecules that initiate neuronal differentiation^37^,^38^. Significantly, it was shown that NO produced by NOS1 is involved in this process^39^. In light of the proposed role of *Mm-antiNos1* RNA in the regulation of NO signalling during neurogenesis, it was very interesting to examine whether neuronal differentiation of Neuro2a cells is associated with specific changes of *Mm-antiNos1* RNA expression. Our results not only provided an affirmative answer to this question but also demonstrate that the changes in the expression of both *Nos1* mRNA and *Mm-antiNos1* RNA observed during differentiation of Neuro2a cells are similar to those, which occur during embryonic neurogenesis. These findings reinforced our belief that *Mm-antiNos1* RNA plays a role in normal neurogenesis through the fine-tuning of *Nos1* gene expression.

It is of interest to note that our previous studies conducted on molluscs have identified a neuronal NAT, which is also transcribed from the non-template strand of a NOS locus^6^. This evolutionary conservatism of the transcriptional profile of NOS-encoding loci observed in very distantly related organisms, such as mammals and molluscs, provides further support for the functional importance of the *Mm-antiNos1* RNA expression.

Finally, recent studies have indicated that in mammals long NATs contribute to the regulation of *NOS2* and *NOS3* gene expression^15^,^16^. However, until now the question regarding whether the *NOS1* related NATs exist, had remained unsolved. The data presented here provide an affirmative answer to the question. We can now conclude that all three mammalian NOS-encoding mRNAs have their antisense counterparts. This suggests an important role for long NATs in controlling NO signalling in mammals.

## Methods

All experimental protocols were performed with the approval of the University of Sussex Ethical Review Committee and in accordance with UK Home Office regulations.

### Animal use and tissue collection

Mice derived from crosses between CBA/Ca and C57BL6/J inbred strains were used. Following humane euthanisation, either the whole brain or the olfactory bulb were rapidly dissected on ice cold PBS and frozen in liquid nitrogen to minimise RNA degradation. Animal husbandry and procedures were followed in accordance with UK Home Office guidelines.

### Northern blot hybridization

Approximately 3 μg of poly(A)^+^ RNA isolated from the mouse brain using Oligotex mRNA Mini kit (Qiagen, Hilden, Germany) were resolved in a 1% denaturing formaldehyde-containing agarose gel and transferred onto NYTRAN-N membrane (Schleicher & Schuell, Keene, NH). Two different single stranded ^32^P-labeled hybridisation probes were generated by asymmetric PCR. The ‘sense’ probe has the same sequence as *Nos1* cDNA whereas the ‘antisense’ probe is complementary to *Nos1* cDNA. Hybridizations were performed at 45 °C in a buffer containing 10% dextran sulfate, 5xSSPE, 5xDenhardt’s solution, 50% formamide, 0.5% SDS, and 100 μg/ml denatured and sheared salmon sperm DNA.

### Verification of the orientation of *Mm-antiNos1* RNA

Total RNA was extracted from the mouse brain by means of Absolutely RNA Miniprep kit (Agilent) and then separated into poly(A)^+^ and poly(A)^−^ fractions by means of Dynabeads oligo-(dT)_25_ (Dynal). The poly(A)^+^ RNA was additionally treated with DNase TURBO according to the manufacturer’s protocol (Ambion). Two cDNA synthesis reactions were carried out on the DNA-free poly(A)^+^ RNA in the presence of SuperScript II reverse transcriptase (Life Technologies). In the first reaction we used primer 1 (5’-CACCCAATTCCTTGTTTCC-3’), which is complementary to *Mm-antiNos1* RNA). In the second reaction we used primer 2 (5’-AACACCCTTGTTACCACAC-3’), which has the same sequence as *Mm-antiNos1* RNA. After completion of the reactions, cDNA primers were removed by means of Chroma Spin-100 columns (Clontech). Purified cDNAs were subjected to 35 cycles of PCR using HotStar Taq DNA polymerase (QIAGEN) and primers 5’-AACACCCTTGTTACCACAC-3’ and 5’-TTGTTTCCTGCAGCCGATC-3’. PCR products were resolved on 1% agarose gel and purified using the QIAquick Gel Extraction kit (QIAGEN). The identity of PCR products was confirmed by DNA sequencing.

### Real-time reverse transcription-PCR

Total RNAs were extracted from individual samples of brain tissue or Neuro2a cultured cells by means of the Absolutely RNA Microprep or Miniprep kits (Agilent Technologies). To remove all traces of genomic DNA the extracted RNAs were treated with DNase TURBO (Ambion). Purified RNAs were copied into cDNAs using the iScript cDNA synthesis kit (Bio-Rad). cDNAs were amplified and analyzed on the Mx3000P real-time cycler (Stratagene) using the Brilliant II SYBR Green QPCR Master Mix (Agilent Technologies). We used primers 5’-TGAGTACCAGCCTGATCC-3’ and 5’-ACGGCCTCTGCCAATTTC-3’ for detection of *Nos1* mRNA, primers 5’-AAAACACCCTTGTTACCACAC-3’ and 5’-AGCTCTTGTCCGTACCAC-3’ for detection of *Mm-antiNos1* RNA and primers 5’-TGTCTCCTGCGACTTCAAC-3’ and 5’-AGCCGTATTCATTGTCATACC-3’ for detection of *GAPDH* mRNA. The identity of all PCR products was confirmed by sequencing. The amount of target transcript, normalized to an endogenous reference (*GAPDH*) and relative to a calibrator was calculated as 2^−ΔΔCT^ where ΔΔC_T_ = ΔC_T_ − ΔC_T(CAL)_. ΔC_T_ and ΔC_T(CAL)_ are the differences in threshold cycles for target and reference (*GAPDH*) measured in the samples and in the calibrator (CAL) respectively.

### Cloning of Mm-antiNos1 cDNA into a mammalian expression vector

Approximately 1 μg of total RNA extracted from the mouse brain was used for cDNA synthesis in a 20 μl reaction using the RevertAid kit (Fermentas) according to the manufacturer’s protocol. 2 μl of the cDNA were added to the amplificaion reaction conducted in the presence of PrimeSTAR GXL DNA polymerase (Takara) and primers 5’-CACCGCTTGTTTCCTAGGAAG-3’ and 5’-CAGGATGAGAAGTGATAAGTAGAG-3’. PCR-generated full-length *Mm-antiNos1* cDNA was gel purified and cloned into pcDNA 3.1 Directional TOPO (Invitrogen). Hereafter we will refer to the recombinant plasmid as pcDNA 3.1/Mm-antiNos1. Successful incorporation and fidelity of the insert were confirmed by DNA sequencing.

### Maintenance, transfection and differentiation of Neuro2a cells

Mouse Neuro2a cells were obtained from the American Type Culture Collection. Cells were cultured in DMEM (Gibco) containing 10% heat-inactivated FCS (PAN Biotech) and penicillin/streptomycin (Gibco) at 37 °C in 95% air, 5% CO_2_.

For transfection experiments, approximately 5 × 10^5^ cells were plated into 60 mm Petri dishes to achieve 50% confluence in 24 hours. The following transfection mixtures were prepared: i) 0.2 μg of pcDNA 3.1/Mm-antiNos1 plus 4.8 μg of wild-type pcDNA 3.1 vector; ii) 1 μg of pcDNA 3.1/Mm-antiNos1 plus 4 μg of wild-type pcDNA 3.1 vector. Cultured cells were then incubated with the transfection mixtures in the presence of Lipofectamine 2000 (Life Technologies) according to the manufacture’s protocol. After 48 hours cells were washed with PBS and collected for real-time RT-PCR and Western blot analysis.

For differentiation assays, Neuro2a cells were plated into 100 mm Petri dishes to reach 25% confluence. The cells were then differentiated by incubating in DMEM containing reduced serum medium (0.1% FCS) and 30 μM retinoic acid (Sigma). After 4 days the cells were washed with PBS and collected for further analysis.

### Western blotting

Protein samples for western blotting were prepared from transfected and non-transfected Neuro2a cells. Cell pellets were resuspended in 200 μl ice-cold homogenization lysis buffer (20 mM Tris-HCl, pH 7.5, 1 mM EDTA, 5 mM DTT, 10% Glycerol, 1% Protease inhibitor and 1% Triton-X-100) and incubated for 15 minutes on a rocker at 4˚C. The samples were then further homogenized using plastic pestles and centrifuged at 13,000 g for an hour at 2 °C. Supernatants were collected and proteins were precipitated with cold (−20 °C) acetone. Precipitated proteins were collected by centrifugation for 5 minutes, air-dried at room temperature and solubilized in 125 mM Tris-HCl, pH 6.8. An equal volume of 2x reducing SDS-PAGE sample buffer (50 mM Tris-HCl, pH 6.8, 2% (w/v) sodium dodecyl sulfate, 10% glycerol, 0.1 M dithiothreitol, 5% β-mercaptoethanol, 0.01% bromophenol blue) was added to solubilized proteins and then the samples were reduced by heating. Equal amounts of proteins were loaded into the wells of the SDS-PAGE gel. The stacking gel contained 4% acrylamide, and the upper and lower halves of the separating gel were composed of 8.25% and 10% acrylamide, respectively. Prestained color markers (Bio-Rad, UK) and a biotinylated protein ladder for horse-radish peroxidase (HRP) detection (Cell Signalling, UK) were used as molecular weight markers. Separated proteins were blotted overnight onto Amersham Hybond-P PVDF membrane (GE Healthcare Life Sciences, USA) using a wet electro-blotting system (Bio-Rad, UK). After protein transfer, the membrane was cut into two sections. The upper section contained proteins with a molecular weight larger than 60 kDa and the lower section contained proteins with a molecular weight below 60 kDa. Both sections were preblocked with 5% non-fat dry milk in TBS containing 0.15% Tween-20. The upper section was then incubated with mouse monoclonal anti-Nos1 sc-5302 antibodies (Santa Cruz Biotechnology, USA) at 1:1000 and the lower section was incubated with mouse monoclonal anti-β-actin antibodies (Sigma, UK) at 1:5000. HRP-conjugated goat anti-mouse IgG at 1:1000 (Cell signalling, UK) was used to detect the bound primary antibodies. HRP conjugates were detected and visualized using the Immobilon western blotting HRP substrate (Millipore, UK). Quantitative densitometric analysis was performed using ImageJ software from National Institutes of Health (USA). Values for the Nos1 expression were quantified relative to the expression of β-actin. Finally, the values for Nos1 expression in transfected Neuro2a cells were normalised to Nos1 protein levels in non-transfected cells.

## Additional Information

**How to cite this article**: Korneev, S. A. *et al.* A novel long non-coding natural antisense RNA is a negative regulator of *Nos1* gene expression. *Sci. Rep.* **5**, 11815; doi: 10.1038/srep11815 (2015).
